# Multiple Modes of Cell Death Discovered in a Prokaryotic (Cyanobacterial) Endosymbiont

**DOI:** 10.1371/journal.pone.0066147

**Published:** 2013-06-18

**Authors:** Weiwen Zheng, Ulla Rasmussen, Siping Zheng, Xiaodong Bao, Bin Chen, Yuan Gao, Xiong Guan, John Larsson, Birgitta Bergman

**Affiliations:** 1 Key Laboratory of Bio-Pesticide and Chemical Biology, Ministry of Education, Fujian Agriculture and Forestry University, Fuzhou, China; 2 Department of Ecology, Environment and Plant Sciences, Stockholm University, Stockholm, Sweden; 3 Biotech Institute, Fujian Academy of Agricultural Sciences, Fuzhou, China; 4 Department of Plant Pathology, Pennsylvania State University, University Park, Pennsylvania, United States of America; University of Lausanne, Switzerland

## Abstract

Programmed cell death (PCD) is a genetically-based cell death mechanism with vital roles in eukaryotes. Although there is limited consensus on similar death mode programs in prokaryotes, emerging evidence suggest that PCD events are operative. Here we present cell death events in a cyanobacterium living endophytically in the fern *Azolla microphylla,* suggestive of PCD. This symbiosis is characterized by some unique traits such as a synchronized development, a vertical transfer of the cyanobacterium between plant generations, and a highly eroding cyanobacterial genome. A combination of methods was used to identify cell death modes in the cyanobacterium. Light- and electron microscopy analyses showed that the proportion of cells undergoing cell death peaked at 53.6% (average 20%) of the total cell population, depending on the cell type and host developmental stage. Biochemical markers used for early and late programmed cell death events related to apoptosis (Annexin V-EGFP and TUNEL staining assays), together with visualization of cytoskeleton alterations (FITC-phalloidin staining), showed that all cyanobacterial cell categories were affected by cell death. Transmission electron microscopy revealed four modes of cell death: apoptotic-like, autophagic-like, necrotic-like and autolytic-like. Abiotic stresses further enhanced cell death in a dose and time dependent manner. The data also suggest that dynamic changes in the peptidoglycan cell wall layer and in the cytoskeleton distribution patterns may act as markers for the various cell death modes. The presence of a metacaspase homolog (domain p20) further suggests that the death modes are genetically programmed. It is therefore concluded that multiple, likely genetically programmed, cell death modes exist in cyanobacteria, a finding that may be connected with the evolution of cell death in the plant kingdom.

## Introduction

Programmed cell death (PCD) is a self-inflicted genetically-based cell death mechanism in eukaryotic organisms, and genetic and cytological studies have led to the identification of pathways and molecular components that underlie this process [1]–[3]. Emerging evidence suggests that PCD may also be operative in prokaryotes, which were previously considered to be immortal unless killed or eaten by predators. PCD may for instance be involved in developmental life cycles and in optimizing adaptations in natural prokaryotic populations subjected to environmental stresses [4]–[6]. Mechanisms that balance life and death are also known to protect against antibiotics and macrophages during bacterial biofilm formation [7].

Studies of prokaryotic PCD have primarily focused on autolysis and in a limited number of bacteria, such as *Echerichia coli* [7]–[9]. Homologs of metacaspases, a family of caspase-like proteins originally described in plants, fungi and protozoa [10] have now been identified in numerous prokaryotic genomes and here we use the term metacaspase for these prokaryotic homologs. These genes were recently shown to have a limited distribution (18%) among the 1400 sequenced prokaryotic genomes [11], although they are widely scattered over the bacterial radiation and particularly frequent among phenotypically complex representatives of Alphaproteobacteria, Deltaproteobacteria and Cyanobacteria. Notably, some of these bacteria hold intriguing evolutionary positions related to the origin of mitochondria and chloroplasts and the evolution of eukaryotes.

The globally widespread cyanobacterial phylum exhibits Gram-negative characteristics, introduced oxygenic photosynthesis, and their evolutionary significance in shaping the biosphere is indisputable [12]. Besides being widespread as free-living entities, cyanobacteria entered pigment-free eukaryotes ∼2500 MYA in a monophyletic event [13], [14], and subsequently evolved into chloroplasts. Through this endosymbiosis, plants conquered global land masses 5–600 MYA ago [12]–[14], and today constitute the largest biomass on Earth. Whereas the identity of the cyanobacterial progenitor of chloroplasts remains unknown, some extant cyanobacteria (genus *Nostoc*) form intimate relationships with plants in a range of divisions [15]. A driving force behind contemporary cyanobacterial-plant symbioses is the ability of the cyanobacterium to fix atmospheric dinitrogen and to fully support a combined nitrogen-independent life-style of the host plant. In the small pteridophyte (fern) *Azolla*, cyanobacteria occupy cavities located within each individual plant leaf [16], [17]. The unique interaction between the partners is reflected in the loss of cyanobacterial autonomy, a consequence of a massive pseudogenization and an eroding genome [18], the first discovered in a plant symbiosis. Moreover, a small cyanobacterial inoculum is kept during the sexual reproduction of the *Azolla* plant using a vertical transfer mechanism that is unique amongst plant symbioses [19], [20]. To date, PCD-like events have been documented experimentally in some free-living cyanobacteria, notably the unicellular *Microcystis* and the filamentous genera *Anabaena* and *Trichodesmium* [4], [21], [22]. Indeed, the genomes of the two latter are by now also known to hold particularly many metacaspase genes, although some may have other roles [11].

To deepen our understanding of the role of death events in prokaryotes in general, we selected the cyanobacterium subject to an endosymbiotic lifestyle in an ancient plant (i.e., *Azolla*) as our model system. We hypothesized that PCD events may underlie some cell developmental features typical of the morphologically complex endosymbiont [16], [17] as well as in the strict regulation of the small cyanobacterial population size inside the plant and during the vertical transfer, and hence in symbiotic homeostasis [18], [20]. Several approaches were optimized and used to monitor cell death events, such as light- and electron microscopy,, following published guidelines to reduce the risk of artifacts [23], [24] as well as biochemical assays and genetic analysis. Our data emphasize the existence of sophisticated cyanobacterial cell death events, and provide structural profiling for cell death as well as important clues for a better understanding of potential PCD processes in prokaryotes. We discuss evolution of PCD in higher life forms, such as plants, in light of our findings.

## Results

In order to search for evidence for the presence of programmed cell death (PCD) in a prokaryote a battery of approaches were used (morphological, biochemical, ultrastructural and genetic), each specific in its own way. The biological test material selected was a cyanobacterium living in symbiosis within leaves of the small water-fern *Azolla microphylla* (from now on *Azolla*) grown under green-house conditions.

### Morphological Criteria

**Figure 1:**
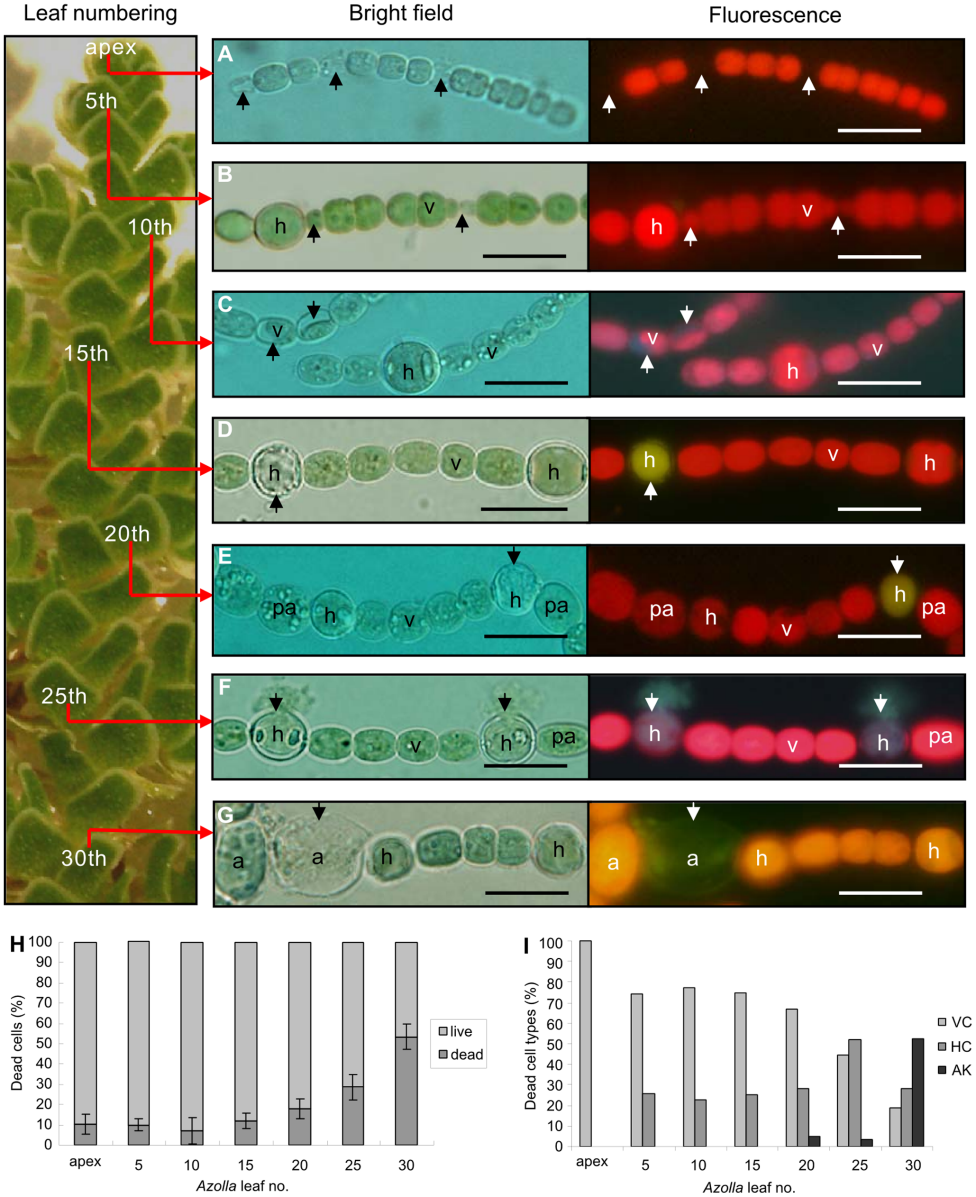
Life and death of an endosymbiotic cyanobacterium. (**A–G**) Cyanobacteria isolated from leaf cavities of various developmental stages of the water fern *Azolla microphylla*. The leaves of the *Azolla* frond (∼1.5 cm long) are numbered along the main plant axis starting at the apex (left panel). Isolated cyanobacteria are visualized using bright field (middle panel) and fluorescence (right panel) microscopy (healthy cells fluoresce red). (**A**) Filaments of small-celled motile hormogonia, functioning as plant colonizing units, at the plant apex. Note the partly lysed cells lacking fluorescence, indicative of dead/dying cells (arrows). (**B**) Heterocystous filament in leaf no. 5. Dead vegetative cells (arrows) characterized by a reduction in cell volume and weak fluorescence. (**C**) Heterocystous filament in leaf no. 10 with dying vegetative cells (arrows), characterized by retraction of the cellular content from the cell wall and vacuolization (faint blue fluorescence). (**D**) Heterocystous filament in leaf no. 15, with a dead heterocyst fluorescing green (arrow). (**E**) Heterocystous filament in leaf no. 20, with a dying heterocyst (arrow) and differentiating pro-akinetes. (**F**) Heterocystous filament in leaf no. 25. The dying heterocysts have ruptured cell walls and are leaking cellular contents (blue). (**G**) Heterocystous filament in leaf no. 30 with a swollen and chlorotic dying akinete fluorescing green. Abbreviations: v, vegetative cell; h, heterocyst; pa, pro-akinete; a, akinete. (**H–I**) Diagram showing the percentage (**H**) and proportion of cell types (**I**) of living and dead cells in the leaf cavities along the main stem of *Azolla* fronds. Abbreviations: v, vegetative cell; h, heterocyst; a, akinete; pa, pro-akinete. Scale bars: 10 µm.

Approximately 25,000 cyanobacterial cells were isolated from leaf cavities of 102 *Azolla microphylla* sporophytes, representing all developmental stages of the plants (Figure 1, left panel), and from 55 sporocarps, the reproductive generation of ferns (Figure S1 and S2). Of these cells, 9,043 were examined under bright field and fluorescence microscopy (filter sets for blue and green light and UV light). In total, 17.16% (1,552 cells) of these cells were considered dead or dying based on the following morphological criteria (middle panel, Figure 1A–G): loss of cell membrane integrity; leaking of cellular content; considerable reduction of cell volume; and chlorotic or swollen appearance with a shrunken cytoplasm, retracting from the cell wall. Moreover, only cells that lacked the bright red autofluorescence (non-affected cells) were defined as dead (right panel, Figure 1). The loss of the autofluorescing pigments chlorophyll a and phycobiliproteins from dying cells results in cells being weakly red or green when excited by green light (550 nm; right panel, Figure 1B–D,E) [25] or blue when excited by UV light (330 nm; right panel, Figure 1F).

Based on these criteria, cyanobacterial cell death events were apparent in all leaf cavities, whereas the proportion and cell types affected varied. The frequency increased with leaf age and peaked at 53.6% of the total cell population in leaf no. 30 (Figure 1H) or older plant parts depending on growth conditions. In recently developed cavities at the plant apex, occupied by cyanobacteria in the small-celled motile hormogonia stage (Figure 1A), about 10% of the cells were dying, while the majority were actively dividing, colonizing new leaf cavities and starting to differentiate heterocysts (Figure S1). In younger leaves with few heterocysts, 77.4% of the total number of dead cells was in the vegetative cell stage (leaf no. 10), while in older leaves the proportion of dead heterocysts reached 51.9% (leaf no. 25), and that of akinetes 52.7% (leaf no. 30) of the total number of dead cells (Figure 1I).

During sexual reproduction of the *Azolla* plant, motile hormogonia at the apex are attracted to megasporocarp initials (Figure S1 and S2) in which they subsequently colonize the inducium chamber. Of the approximately 631±49 cyanobacterial cells that initially colonized the smaller female megasporocarp about 21% (135 cells) were sacrificed before its maturation, while the rest of the cyanobacterial inoculum was transferred to the next plant generation. The larger male microsporocarps were initially colonized by hormogonia, but unlike in the female megasporocarp all cyanobacterial cells disappeared during the microsporocarp maturation (Figure S2).

### Biochemical Criteria

**Figure 2:**
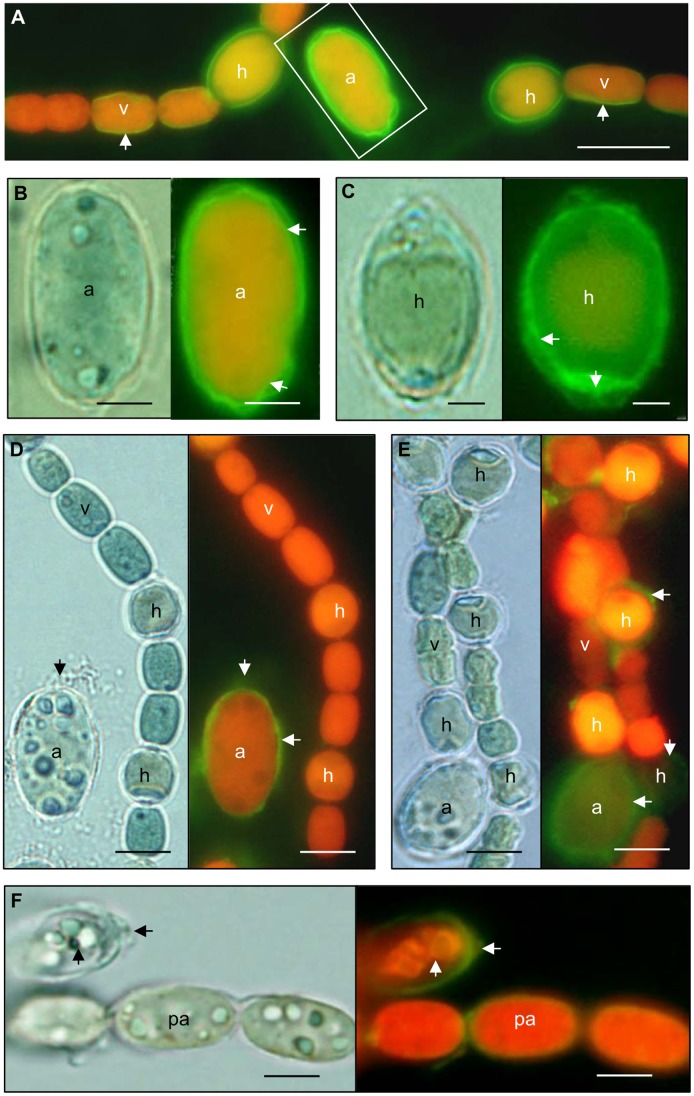
Early stages of cyanobacterial cell death visualized by the Annexin V-EGFP assay. The Annexin V-EGFP assay emits green fluorescence when phosphatidylserine (PS) is exposed at the outer membrane. (**A**) Cyanobacterial filaments (leaf no. 20) showing a distinct green Annexin V-EGFP signal at the vegetative cell plasma membrane (arrow) and on the external envelope of the heterocysts and akinete. (**B**) Magnification of the granulated akinete (boxed in A) under bright field LM (left) and fluorescence microscopy (right), showing the Annexin V-EGFP label (arrows). (**C**) A thick-walled heterocyst under bright field LM (left) and fluorescence microscopy (right), showing Annexin V-EGFP label and a ruptured cell wall (arrows). (**D**) A non-affected cyanobacterial filament (leaf no. 25) and a large granulated akinete displaying an Annexin V-EGFP signal (right) and leakage of cellular contents (arrows). (**E**) Cyanobacterial filaments (leaf no. 30) with green Annexin V-EGFP fluorescence (arrows) at heterocysts and the akinete. (**F**) Pro-akinetes in a sporocarp with an Annexin V-EGFP signal and a retracted cytoplasm (arrow). Abbreviations: a, akinete; h, heterocyst; v, vegetative cell. Scale bars: A-B 5 µm; C 4 µm; D-F 10 µm.

In order to examine whether the cell death events observed using morphological criteria were related to PCD, the cyanobacterial population within the *Azolla* plants were subject to biochemical tests. To this end two assays were used: the Annexin V-green fluorescent protein (Annexin V-EGFP) assay, which registers externalized phosphatidylserine (PS) at the plasma membrane, and is used as an indicator of early apoptosis [23], and the TUNEL assay, which incorporates fluorescein-dUTP into the ends of fragmented DNA to detect DNA cleavage and condensation, and is used as an indicator of late apoptotic events [26]. In both assays, the cyanobacterial cells stained coincided approximately with cells identified as dying/dead cells using the morphological criteria. Annexin V-EGFP fluorescence was apparent in cyanobacterial cells from leaf cavities and developing sporocarps (Figure 2). The Annexin V-EGFP binding was localized at the cell membranes in all cyanobacterial cell types, i.e., vegetative cells, akinetes and heterocysts (Figure 2A–C). Label was also detected in dying and disrupted akinete initials leaking their cellular content, and the number of heterocysts and akinetes exhibiting signs of apoptosis was higher in older leaves (Figure 2D–E). Even some pro-akinetes within the developing sporocarps were characterized by cytoplasm condensation and Annexin V-EGFP labeling (Figure 2F).

**Figure 3:**
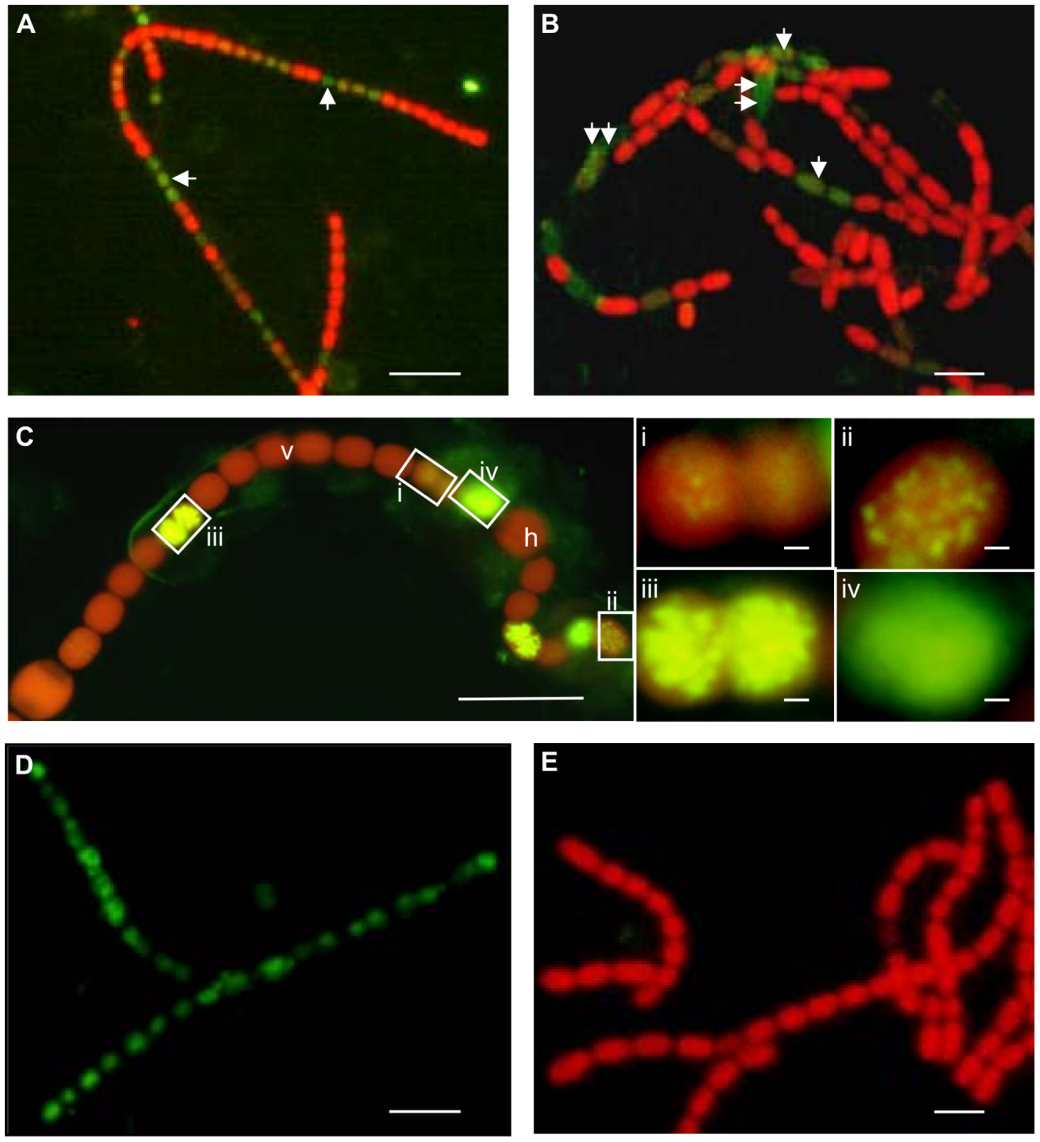
Later stage cyanobacterial cell death visualized by the TUNEL assay. The TUNEL assay emits green fluorescence in response to DNA fragmentation. (**A**) Hormogonium filaments at the plant apex, with groups of cells showing a positive TUNEL reaction (arrows). Non-affected cells exhibit red autofluorescence. (**B**) Chains of pro-akinetes in a megasporocarp which exhibits green fluorescence in apoptotic-like (arrow) and necrotic-like (double arrows) cells, the latter showing leaking cytoplasms. (**C**) A heterocytous filament (leaf no. 15) showing green TUNEL fluorescence in vegetative cells entering the later stages of the cellular death process: i) ∼10% of the DNA is fragmented (dots) and faintly stained in green-red, due to the red autofluorescence of the background pigmentation; ii) ∼50% of the DNA is stained and the fluorescing dots are larger; iii) ∼80% of the DNA is intensively stained; and ix) the DNA fragmentation is complete and the green fluorescence smeared (i.e., the cells are dead). (**D**) Positive control: homogonium filaments pre-treated with DNase I show a positive TUNEL reaction in all cells. (**E**) Negative control: chains of pro-akinetes with all cells displaying red fluorescence after omitting TdT, which detects the nicked DNA. Scale bars: A-E 10 µm; i-iv in **C** 1 µm.

The TUNEL assay provides biochemical evidence for the existence of later stages in the PCD pathway. TUNEL fluorescence signals were found in hormogonia, vegetative cells, heterocysts and akinetes, of both leaf cavities and developing sporocarps (Figure 3). The green positive TUNEL signal was often detected in groups of two to four vegetative cells along the filaments in leaf cavities, and in pro-akinetes within developing megasporocarps (Figure 3A–B). Based on cell structure, cell volume and TUNEL fluorescence signal intensity, the dying cells were grouped into apoptotic-like and necrotic-like cells (Figure 3B), the latter characterized by cytoplasmic leakage. Intermixed with non-affected cells, cells with a range of TUNEL-positive signal strengths were identified within the same filament. Based on the intensity and DNA fluorescence distribution, the TUNEL assay suggested four different PCD phases (Figure 3C): i) initial phase in DNA fragmentation, weak fluorescence and few scattered patches of fluorescein-dUTP-incorporated DNA (spots); ii) intermediate phase, more pronounced DNA fragmentation, larger and additional fluorescent spots; iii) late phase, advanced DNA fragmentation, a strong fluorescent TUNEL signal; and iv) final phase, fully degraded DNA, characterized by a strong smeared TUNEL signal. Morphological changes were not visible (at the LM level) at steps i) to iii), whereas plasma membrane rupture and leakage of cellular inclusions were significant for cells at step iv). The data were verified by examining cells pre-treated with DNase I which exhibited green fluorescence while green fluorescence was lacking when the TdT enzyme was omitted (Figure 3D–E).

### Cytoskeleton Criteria

**Figure 4:**
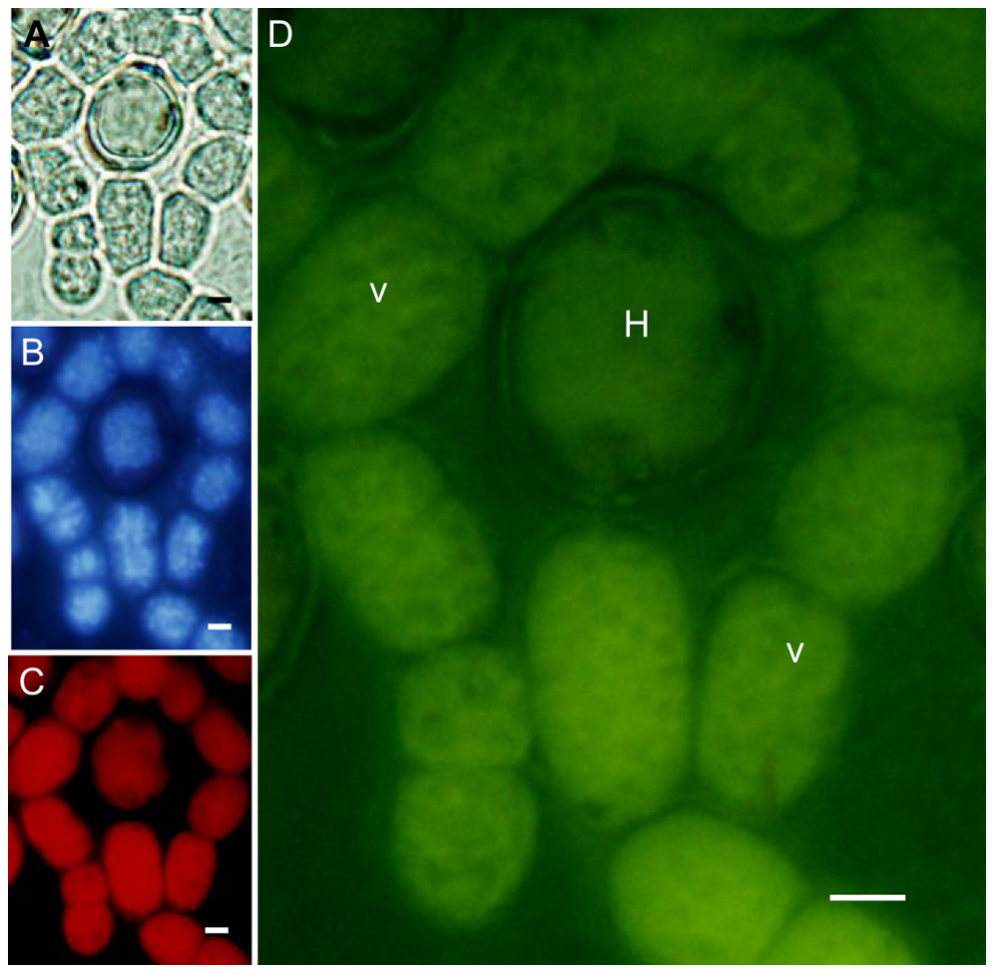
Actin distribution pattern in healthy cyanobacteria. (**A**) Bright field light microscopy of vegetative cells not affected by cell death. (**B**) DAPI (DNA) stained cells (UV filter set). (**C**) Autofluorescence by phycobiliproteins (green light filter set). (**D**) The cellular meshed network of an actin-like cytoskeleton is visualized after staining with phalloidin and is apparent as a green fluorescence in healthy vegetative cells (blue light filter set). V, vegetative cells; H, heterocyst, Scale bars: 1 µm.

**Figure 5:**
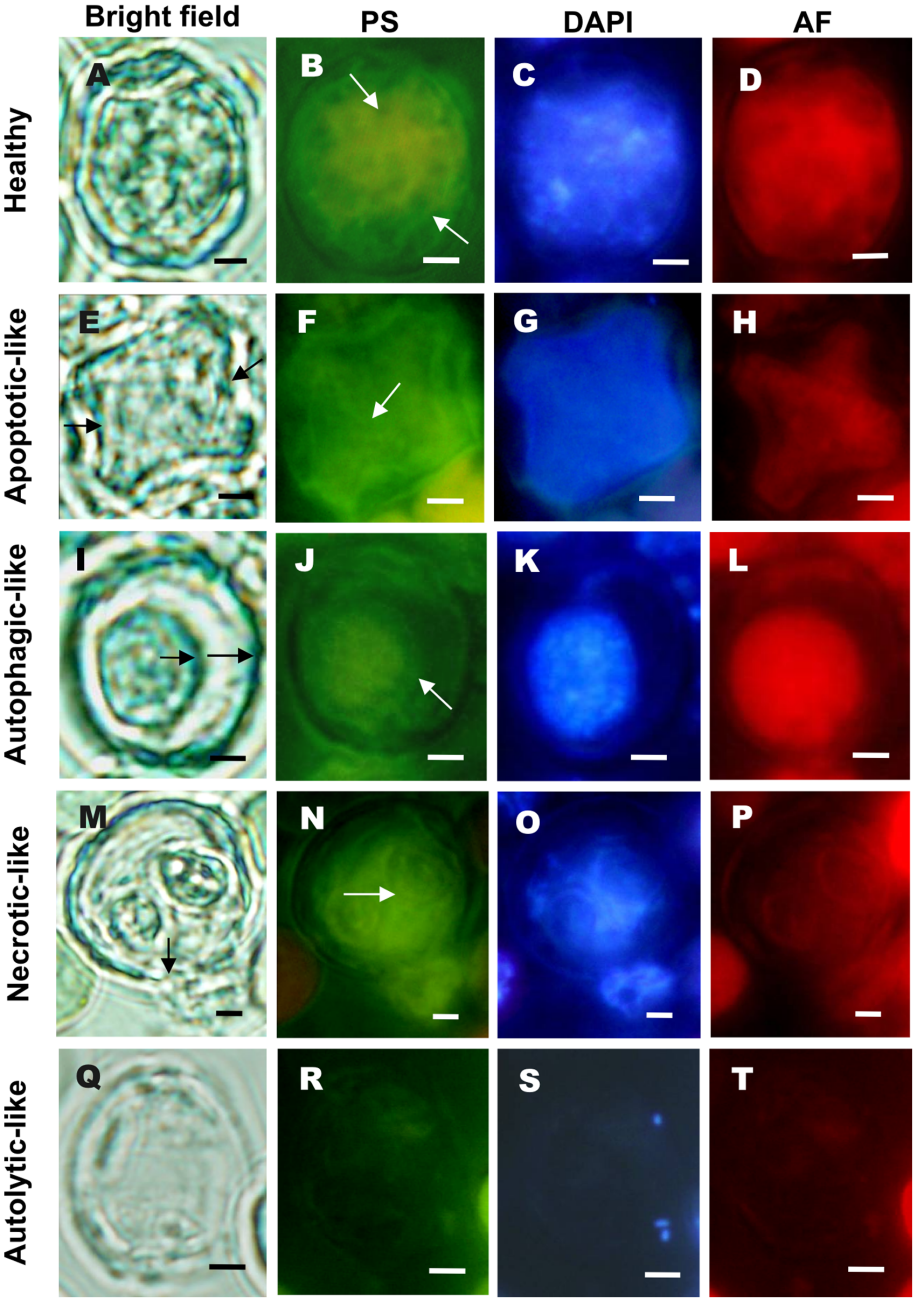
Cellular actin patterns in heterocysts passing through four modes of cell death. (**A–D**) Healthy heterocyst displaying phalloidin stained (PS) actin seen as a meshed network covering the cell (arrows), and a strong blue and red fluorescence of DNA (DAPI) and autofluorescing (AF) photosynthetic pigments, respectively. (**E–H**) An apoptotic-like heterocyst with a shrunken cytoplasm (long arrows) and a polygonal partly disorganized actin pattern (short arrow) and weaker DAPI- and autofluorescence. (**I–L**) An autophagic-like heterocyst with a retracted cytoplasm but with an intact plasma membrane (short arrow) and a condensed but mostly retained actin pattern (long arrow) and strong DAPI- and autofluorescence. (**M–P**) A necrotic-like heterocyst with a disruptered cell wall and plasma membrane (black arrow) resulting in leakage of cellular contents (DNA and pigments), and a more or less disrupted actin (long white arrow). (**Q–T**) An autolytic-like degraded heterocyst lacking most cellular inclusions including the actin network. A, E, I, M and P, bright field; B, F, J, N and R, phalloidin staining; C, G, K, O and S, DAPI-DNA staining; D, H, L, P and T, pigment autofluorescence. Scale bars: 1 µm.

Next, a link between the distribution of the cyanobacterial cellular cytoskeleton [27] and cell death modes was examined using a FITC-phalloidin, a stain which specifically binds to F-actin of the cytoskeleton of eukaryotes. A bright green fluorescing actin-like network was apparent in both vegetative cells and heterocysts (Figure 4 and 5B), at the same time showing a bright red autofluorescence and a strong blue DAPI (DNA) stain, the two latter characteristic of healthy cells. Heterocysts with features of apoptosis, i.e. invaginated but intact cell wall and plasma membrane, still fluoresced green but lacked the well-organized meshed network of actin and exhibited weaker autofluorescence and DAPI staining (Figure 5E–H). Heterocysts with characteristics of autophagy, such as intact plasma membrane but retracted cytoplasm, still retained actin-like structures as well as fluorescing photosynthetic pigments and DNA (Figure 5I–L). A disrupted organization of the actin-like network was found in heterocyst which showed necrotic features, such as swollen cells, ruptured plasma membranes and leakage of cellular contents (Figure 5M–P). Heterocysts exhibiting autolytic characteristics lacked the actin-like structure and most cellular components (Figure 5Q–T). Together these data suggest that the cyanobacterium possess an actin-like cytoskeleton, which shows a reorganization and depolymerization during cell death which reminds of eukaryotic PCD.

### Ultrastructural Criteria

**Figure 6:**
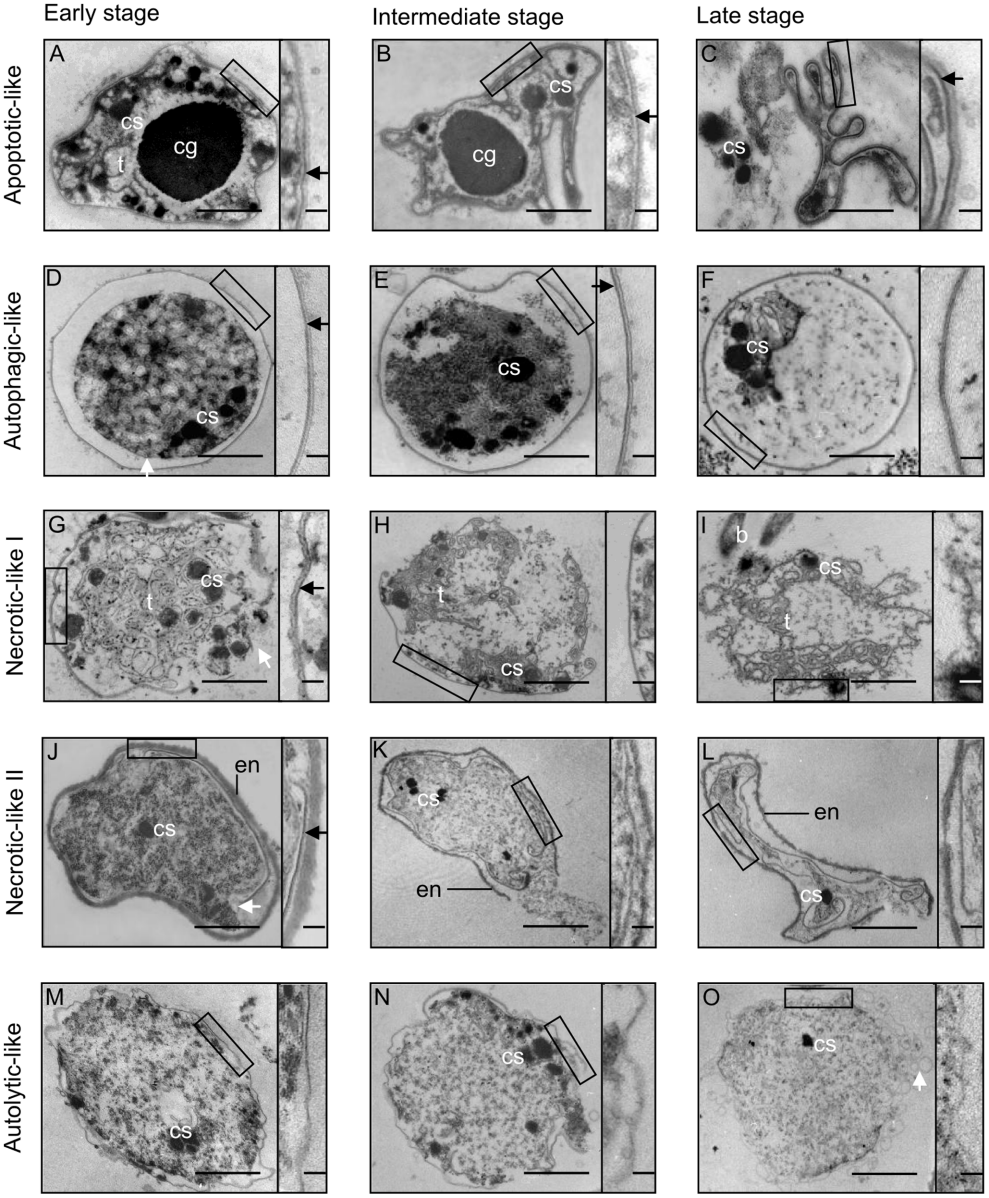
Multiple cell death modes in the cyanobacterium visualized by TEM. Four death modes are illustrated at an early, intermediate and late stage. Figures S3, S4 and S6A depict the ultrastructural appearance of healthy cyanobacterial cell types. The boxed and magnified cell wall area in the TEM micrographs is given to the right of each micrograph. (**A–C**) *Apoptotic-like cell death mode*: (**A**) Cell shrinkage commences and the cellular content is slightly reduced. (**B**) Cytoplasmic shrinkage continues and the cell shape is irregular and invaginated, while the cell wall and cytoplasmic inclusions remain intact. (**C**) Dendritic-like cell morphology and loss of cellular contents are apparent at the later stage, but the cell wall, including the peptidoglycan layer, is still intact (arrow). Peptidoglycan layer intact (arrows). (**D–F**) *Autophagic-like cell death mode:* (**D**) The cell is swollen, while the cytoplasm starts to condense and undergo vacuolization. The outer membrane and peptidoglycan layer are intact. (**E**) The plasma membrane ruptures and continued cytoplasm degradation. The outer membrane and the peptidoglycan layer are still intact (arrow). (**F**) The outer cell shape is retained, while the cellular content and the peptidoglycan layer disappear. (**G–I**) *Necrotic-like cell death mode*, *type I* (affects vegetative cells): (**G**) The cell shape is distorted, the cell wall disrupted and the cellular content released. The peptidoglycan layer is preserved (arrow). (**H**) Continued disintegration of cellular contents and disappearance of the peptidoglycan layer. (**I**) Only partial membranes (thylakoids) remain at this stage. (**J–L**): *Necrotic-like cell death mode, type II* (affects akinetes and heterocysts): death progression as for type I above, but the outer cell envelope of the cells remains until the late stage. (**M–O**) *Autolytic-like cell death mode:* (**M**) The degrading cytoplasm is surrounded by a unit membrane only, and no peptidoglycan layer. (**N**) The membrane continues to dissolve, as does the intracellular content. (**O**) The membrane vesiculates and the cytoplasm disintegrates and leaks out. Abbreviations: b, bacterium; cg, cyanophycin granule; cs, carboxysome; en, envelope; t, thylakoid membrane. Scale bars: A-O: 1 µm; in boxed area 50 nm.

To complement the Annexin V-EGFP and TUNEL assays, which do not discriminate between various PCD cell death modes: apoptotic, autophagic, necrotic and autolytic cell death [28], transmission electron microscopy (TEM) analysis was performed on cyanobacterial cells in *Azolla* leaf cavities and megasporocarps. A total of 940 ultrathin sections and 3,950 cyanobacterial cells, representing a random distribution of cells along the approximately 80 *Azolla* plants tested, were analyzed. Among these, 3,219 cyanobacterial cells appeared healthy showing intact cellular features at the ultrastructural level (see Figure S3, S4 and S6A), whereas the remaining ∼18.5% (731) were dying or dead (Figure 6 and S3i–vi). Hence, and not surprising, detailed TEM analyses lead to a somewhat higher percentage of recognized dead cells compared to lower resolution LM analyses (17.2%).

Closer inspection of the dying cells at the ultrastructural level revealed the existence of multiple cell death modes in the cyanobacterium. These included an apoptotic-like cell death mode in vegetative cells (Figure 6A–C; Figure S4F), characterized by cell shrinkage and thylakoid membrane disintegration at an early stage, while cell walls (membrane/peptidoglycan layer) remained intact and some inclusions, such as carboxysomes and the large cyanophycin granules (N-storage), were retained. The apoptotic-like cell death mode was observed in vegetative cells, heterocysts and akinete initials in developing sporocarps (Figure S3 and S4). The autophagy-like cell death mode was characterized by an intact cell wall system, but a gradual condensation and degradation of the cytoplasm and high levels of vacuolization (Figure 6D–F; Figure S4). This cell death mode affected vegetative cells, pro-heterocysts and mature heterocysts (Figure S3 and S4). In the necrotic-like cell death mode, the cell wall was ruptured at an early stage, resulting in leakage of cellular content. Based on the membrane systems, this cell death mode could in turn be divided into two subtypes: I, characteristic of vegetative cells, the intracellular components were dispersed into the extracellular space, while part of the membrane system remained intact until the late stage (Figure 6G–I; Figure S4); II, characteristic of the thick-walled akinetes, a delay in the leakage of the degraded cytoplasm was apparent (Figure 6J–L). The necrotic-like cell death mode was observed in leaf cavities and sporocarps, and in all cell types (Figure 6G–I,J–L; Figure S3 and S4). Finally, in the autolytic-like cell death mode, the peptidoglycan layer of the vegetative cells lysed at the early stage and the cytoplasm disintegrated gradually and vesicles formed at late stages (Figure 6M–O; Figure S3 and S4). Autolytic-like cell death was the case in vegetative cells (Figure 6M–O) and heterocysts (Figure S3). These cell death modes occurred independently of plant age and plant organ.

**Figure 7:**
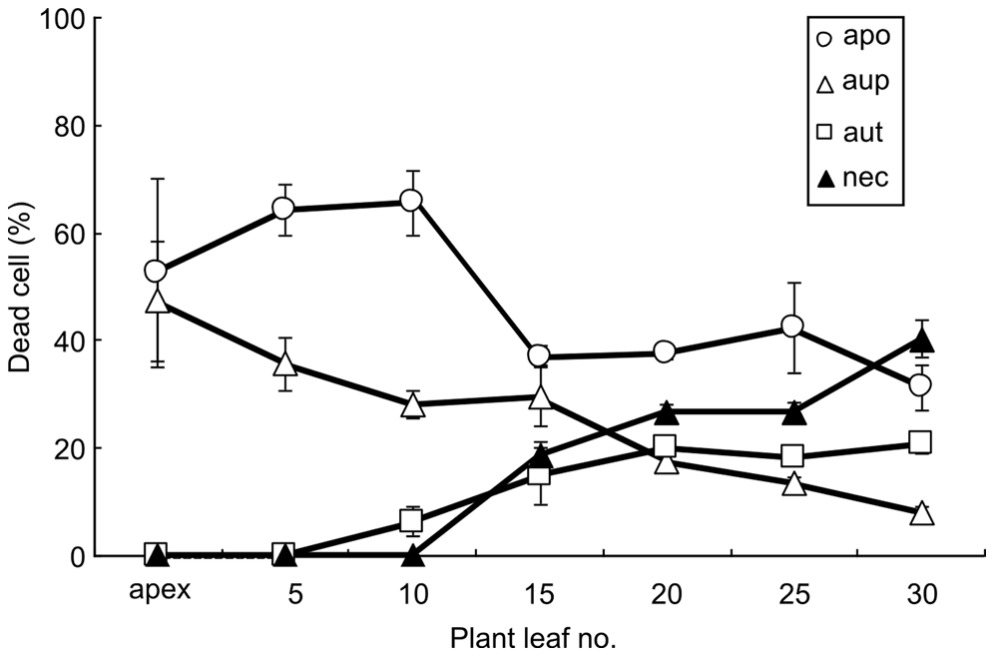
Cyanobacterial cells death modes along the plant developmental gradient. The apoptosis- and autophagy-like death modes dominate the initial plant developmental stages, while the autolysis- and necrotic-like dominate at later developmental stages. aup: autophagy-like; ap:, apoptosis-like; aut: autolysis-like; nec: necrotic-like.

Interesting shifts in the cyanobacterial death modes in relation to the developmental stage/age of the plant was also apparent (Figure 7). Clearly, apoptotic- and autophagic-like modes operate and dominate in young plant parts (apex to leaf no. 15), while necrotic- and autolytic-like events appear at later developmental stages and dominate in cyanobacteria of older plant leaves.

### Stress Enhanced Cell Death

**Figure 8:**
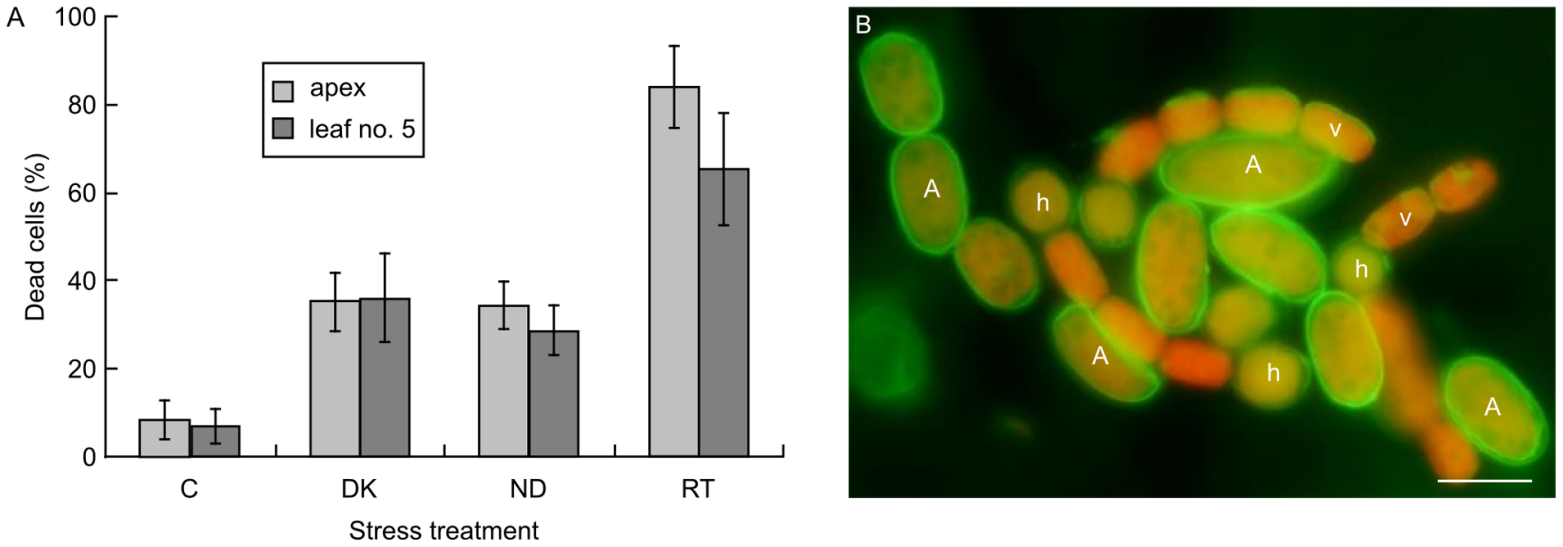
Stresses enhance cell death in the cyanobacterium. (**A**) Percentage of cyanobacterial cells affected by the various death modes identified in relation to two plant developmental stages. (**B**) Cyanobacterial filaments (leaf no. 13) showing a strong Annexin V-EGFP signal in most cells and in all cell types, indicating a strong enhanced cell death in the majority of cells. C, control cells; DK, dark treated; ND, nutrient deprived; RT, radiation treated. v, vegetative cells; h, heterocysts; A, akinetes. Scale bar: 10 µm.

Next, *Azolla* plants were subjected to selected stresses (darkness, nutrient deprivation and γ-radiation). We hypothesized that if PCD is in action in the cyanobacterium, stresses would enhance the process, as shown for other organisms [2]. In total, 1,004 cells from the plant apex (leaf no. 1) and leaf no. 5 were examined by TEM and LM. All stress treatments enhanced distinct morphological alterations and in particular in cyanobacterial cells residing at the plant apex. Dead/dying cells were found at a 3–10 fold higher frequency under stress than under non-stress conditions (Figure 8A). Dark treatment (5 days) lead to an autophagic-like death mode (Figure S5), first cytoplasmic condensation and plasma membrane preservation, then cytoplasmic disorganization followed by vacuolization. This suggests that the cyanobacterium may be subject to PCD. It is also apparent that cyanobacteria at the apex depend on their own photosynthesis, while they live heterotrophically in more mature leaf cavities [16]. Annexin V-EGFP (Figure 8B) and phalloidin-FITC staining (data not shown) of cyanobacterial cells in *Azolla* plants subject to nutrient deprivation (N-depletion) verified that a substantial proportion (32.1%) of the cells examined were negatively affected. Using γ-radiation, at 40 and 80 Gy, enhanced the cyanobacterial cell death frequency substantially, to 83.7% and 65.3% for cyanobacteria at the apex and in leaf no. 5, respectively (Figure 8A). At the ultrastructural level a clear cytoplasmic condensation and a reduction in cellular volume was apparent indicative of necrotic-like and autolytic-like cell death modes (Figure S6).

Finally, we examined the ultrastructure of dead cells and cell debris in leaves and sporocarps (Figure S7). When the peptidoglycan layers of the gram negative cell wall were digested, numerous vesicles appeared at the original location of the peptidoglycan layer (Figure S7E,F). Some of the dying/dead cells were still at this late stage connected to healthy-looking cyanobacterial cells (in both leaf cavities and megasporocarps) and the majority was in close proximity to bacteria. To what extent the cyanobacterium undergoes auto-digestion and/or digestion by adjacent bacteria is however an open question (Figure S7A–C).

### Molecular Criteria

**Figure 9:**
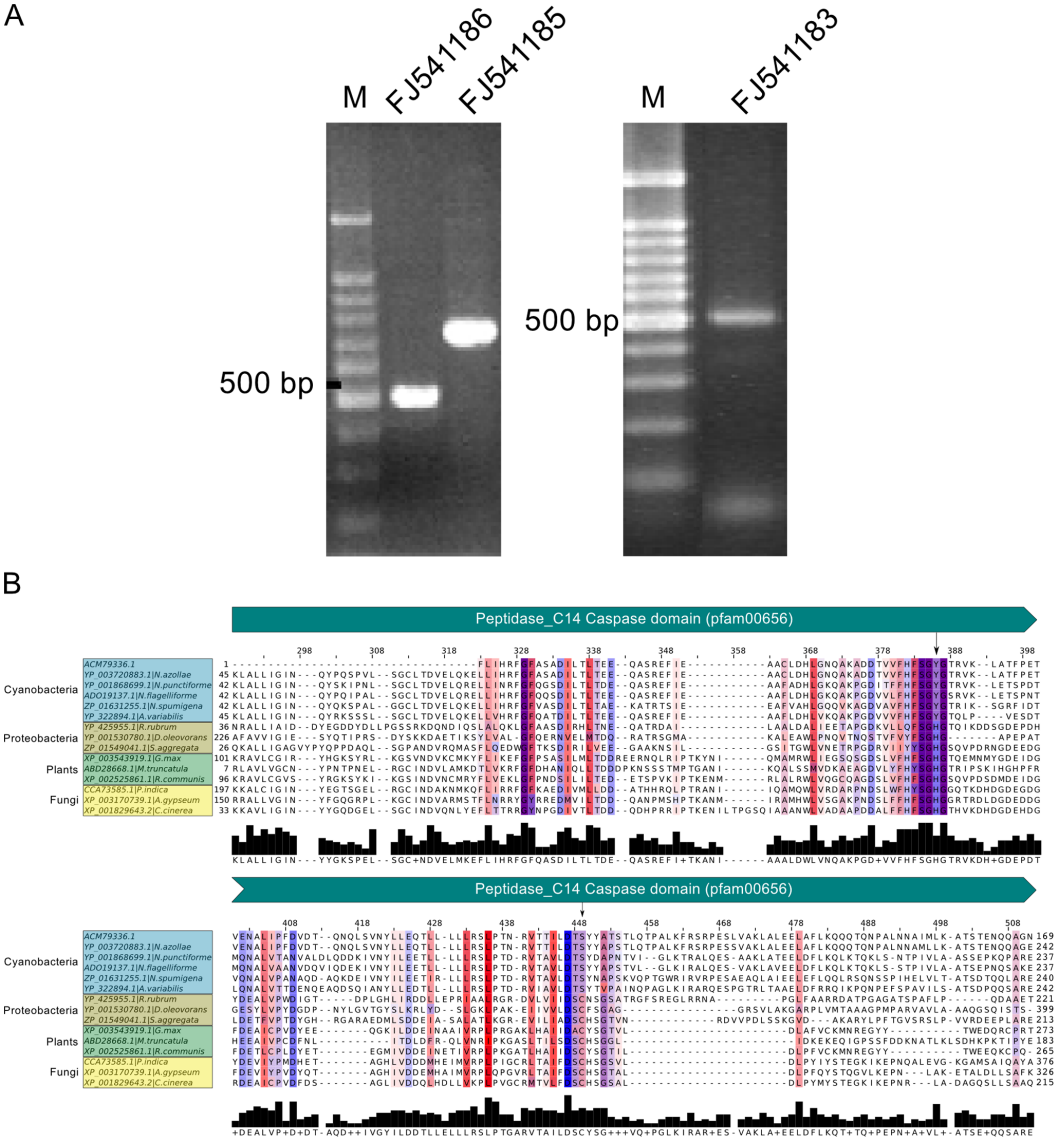
Amplification of cyanobacterial genomic DNA and sequence alignments. (**A**) PCR products FJ541186, FJ541185 and FJ541183 amplified from cyanobacterial DNA with primer pairs cas203, cas202 and HCW3/4, respectively (Methods). M, molecular weight. (**B**) Multiple sequence alignment of the partial protein sequence ACM79336.1, the predicted translation product of FJ541186, and significant homologs in cyanobacteria, plants and fungi. The numbers above the alignment and in the margins denote alignment position and residue position ranges for each sequence, respectively. The modal consensus is shown below the alignment. Residue color indicates hydrophobicity (from red [hydrophobic] to blue [hydrophilic]) and the shading intensity of a column is proportional to conservation (at 30 percent cutoff). The conserved caspase domain (pfam00656) spans this region of the alignment. The conserved catalytic His-Cys dyad is replaced with Y-S in the cyanobacterial sequences (indicated by arrows).

To obtain molecular information related to PCD in the endosymbiotic cyanobacterium of *Azolla*, the primer pair cas202 and cas203 were designed based on encoding sequences for conserved catalytic subunit p20 of the apoptotic enzyme caspase (in GenBank accession no. CP000117). Two fragments (754 bp and 508 bp) were amplified from genomic DNA isolated from the cyanobacterium of *Azolla* (Figure 9A), and the sequences were deposited in GenBank with accession numbers FJ541185 and FJ541186, respectively. A BLASTN search showed that FJ541185 and FJ541186 are 99% identical (99% query coverage) to the C-terminal (position 968–1707) and N-terminal (position 221–727) of a “caspase catalytic subunit p20” gene (locus tag Aazo_1578) in the sequenced genome of ‘*Nostoc azollae*’ 0708, the cyanobiont of *A. filiculoides* [18]. This gene in ‘*Nostoc azollae*’ 0708 was identified as a metacaspase [11], [20]. The translated amino acid sequence of FJ541186 (GenBank accession ACM79336) was used as query in a BLASTP search, which identified several homologs in bacteria (including cyanobacteria, proteobacteria and nitrospirae) as well as in plants and fungi (see Table S1 online). The majority of hits are annotated as “caspase”, “metacaspase” or “caspase catalytic subunit p20”, and all significant hits in completed genomes were previously identified [11]. This indicates that ACM79336 belongs to the superfamily of caspase-like proteases that was previously described as the “metacaspases” in plants, fungi and some bacteria [29]. Figure 9B shows a multiple sequence alignment of ACM79336 with representative homologs in cyanobacteria, proteobacteria, plants and fungi (see also Table 1). The caspase domain (Pfam id pf00656) spans the entire length of ACM79336.1 and is also present in the homologous sequences (Figure 9B). The alignment shows that the catalytic His-Cys dyad common to caspases is replaced with Tyr-Ser in the cyanobacterial sequences. This substitution has been reported previously for cyanobacteria [30] and is the most common type of amino acid substitutions found in bacterial metacaspases [11]. In the protozoan *Trypanosoma brucei*, a Cys-Ser substitution in the active site is known to inactivate some cysteine proteases [31]. However, the alternative enzymatic roles performed by metacaspases holding this (or other) substitution(s) remains to be established in prokaryotes and in protozoa.

**Table 1:** Comparison between cell death modes in animals, plants and cyanobacteria.

Organism	Death mode	Shape/volume	Membrane	Cytoplasm/organelle	Enzyme	DNA	Programmed
Animal	Apoptosis	Rounding up	Intact	Preserved or minor changes	Caspase	Fragmentation	Yes
		Shrinkage	PS exposure	Condensation			
		Reduction ofvolume	Blebbing				
Plant	Apoptotic-like	Reduction ofvolume	Blebbing	Condensation	Caspase-like	Fragmentation	Yes
Cyanobacteria	Apoptotic-like	Shrinkage	Intact	Minor changes	Metacaspase	Fragmentation	Yes
		Reduction involume	PS exposure	Leakage			
Animal	Autophagy	No obvious change	Intact	Au-vacuoles^a^	ATG family	Fragmentation	Yes
			PS exposure	Degradation of organelles			
Plant	Vacuolar celldeath	No obviouschange	Nuclear envelope disassembly	Formation of lytic vacuoles	Hydrolases	Fragmentation	Yes
		Empty corpsesfinally	Rupture of tonoplast	Degradation of organelles			
Cyanobacteria	Autophagy-like	Rounded up	Intact	Condensation and vacuolization		Fragmentation	Yes
		Empty membrane					
		Surrounded bycorpses	PS exposure	Degradation of inclusions			
Animal	Necrosis	Overall brokencells	Rupture of OMand PM	Swelling of organelles	Loss of ATP	Degradation	ND^b^
				Leakage to extracellular space			
Plant	Necrosis	Overall brokencells	Rupture of OM	Swelling of organelles	Loss of ATP	-^c^	-
Cyanobacteria	Necrosis-like	Overall brokencells	Rupture of OMand PM	Swelling/non-swelling	-	-	-
				Leakage to extracellular space			

Definitions of PCD modes according to reference 24, and 1, 4, 6–8, 23 and 37.

a) Au-vacuoles, autophagy vacuoles;

b) ND, Not determined;

c) -, unknown.

Using primer Hcw3/4, which was based on the encoding sequence of cell wall hydrolase of *Anabaena* PCC 7120 (GenBank accession no. AF216288), a fragment (506 bp) was amplified and the sequence was deposited in GenBank under accession no. FJ541183. Our BLASTN analysis also showed that FJ541183 is 99% identical (96% query coverage) to an N-acetylmuramoyl-L-alanine amidase/autolysin gene (locus tag Aazo_0861) in the genome of ‘*Nostoc azollae*’ 0708. A BLASTP search of the translated amino acid sequence of FJ541183 (GenBank accession ACM79334) identified 42 homologs (Table S2 online). All identified homologs are from cyanobacteria, both unicellular and filamentous/multicellular species.

## Discussion

Our data collectively suggest that several programmed cell death events may take place in the life cycle of a prokaryotic cyanobacterium that exhibits advanced developmental and adaptive capacities, as exemplified here in a cyanobacterium adjusting to the highly stringent and stressful conditions offered inside a eukaryotic host. This conclusion is based on the findings that i) the cell death events identified (cell lysis and degradation) operate in a photosynthetic prokaryote intimately associated with a eukaryotic host; ii) four distinct cell death modes are identified, apoptotic-like, autophagic-like, necrotic-like (type I and II, depending on cell type) and autolytic-like, which are similar in several respects to PCD events in eukaryotes [2] (Table 1); iii) the cyanobacterial cell death events may be genetically underpinned as suggested by the presence of autolysin and metacaspase, the latter supported by the rich metacaspase gene pool (with conserved p20 and Cys-His dyads similar to those of eukaryotic caspases) recently found in prokaryotes, and particularly many in developmentally complex cyanobacteria [11], [30]; iv) cell death events are not restricted to photosynthetic vegetative cells, but also take place in heterocysts and resting akinetes, and are potentially therefore involved in differentiation of cells; and that v) these cell death events occur in cyanobacteria irrespective of their location in various plant organs (leaves and sporocarps) and host plant developmental stage (vegetative growth and sexual reproduction), although the relative proportion of the various death modes varied with plant age. Finally, as external stress factors (darkness, nutrient deprivation and ionizing radiation) enhance cell death further supports the existence and potentially, a functional importance, of PCD events in this cyanobacterium. Hence, our data confirm and considerably extend earlier studies on experimentally verified PCD events of some free-living cyanobacteria [4], [21], [22] and show, for the first time, that autophagic-, autolytic- and necrotic-like PCD events may take place in a cyanobacterium.

The finding that all cyanobacterial cell types (i.e., vegetative cells, hormogonia, heterocysts and akinetes) responded positively to Annexin V-EGFP staining suggest that all can pass early stages of apoptotic-like and autophagic-like PCD, and ahead of any visible phenotypic changes [32], [33]. The TUNEL assay further verified the potential existence of later-stage PCD events and progressive DNA fragmentation, yet another hallmark of PCD. This assay suggests that cell death events in cyanobacteria do not occur in a chaotic manner, but represent a regulated process. The distinct phenotypic features identified (cell shrinkage, membrane integrity, cytoplasm preservation, PS exposure and DNA fragmentation) match criteria recently defined for PCD in eukaryotes [24].

Studies have demonstrated that alteration in the actin cytoskeleton is not just a consequence of PCD, but may play a functional role in the initiation and regulation of PCD [34], [35]. We here provide the first account of a link between actin dynamics and four cell death modes in a cyanobacterium, based on different F-actin distribution patterns and polymer levels (e.g. actin stabilization, depolymerization and degradation). The data coincide with findings of cytoskeletons in cyanobacteria [27], [36] and that actin depolymerizes at early stages of apopotosis while it is preserved largely during middle-later stage autophagy. An intact cytoskeleton network stabilizes cellular conformations, while polymerization of actin destroys the original morphology, resulting in the irregular appearance of the apoptotic-like cyanobacterial cells. Actin alternations may therefore be used as an indicator of potential cyanobacterial PCD modes.

Metacaspases (caspase-like proteases) are known to play a crucial role in PCD in plants, fungi and unicellular eukaryotes [29], and a metacaspase was recently found to be active in cell death events of the cyanobacterium *Trichodesmium* sp. [22]. Our identification in the cyanobacterium of genes that encode the conserved catalytic subunit (p20) of caspases, combined with the discovery of a gene encoding N-acetylmuramoyl-L-alanine amidase, which catalyzes cell-autolysis, provides support for the existence of PCD pathways in this cyanobacterium. Genomic surveys of 33 cyanobacterial [30] and more recently 1,463 prokaryotic [11] genomes, revealed the existence of 58 and 671 putative metacaspase genes, respectively, including a metacaspase in the recently sequenced *Azolla* endosymbiont [18]. Genes involved in autophagy or necrosis were not detected in the *Azolla* endosymbiont genome using primers based on conserved domains of some important eukaryotic proteins, such as Atg4, Atg5, microtubule-associated protein1 light chain3 (LC3) [23], and receptor-interacting serine/threonine-protein kinase1 (RIPK1), proposed to be involved in necrosis [2]. Either the similarity between the eukaryotic and prokaryotic genes is too low, or evolutionary homologs of these genes are absent in prokaryotes. However, autophagy and apoptosis, and even necrosis in some cases, may be regulated by the same factors, and 13 proteins have been identified that have dual roles in autophagy and apoptosis [2], [37], [38].

Although cyanobacteria as opposed to eukaryotic cells lack membrane-bound organelles, they do contain elaborate membrane systems and subcellular inclusions; for instance photosynthetic thylakoids, cyanophycin and carboxysomes. Still, a comparative evaluation of PCD events in animals, plants and cyanobacteria, based on nomenclature recommendations by Kroemer and co-workers [24], suggests a number of apoptotic-like parameters to be shared between cyanobacteria and eukaryotes as summarized in Table 1. This supports the existence of a cyanobacterial apoptosis process. One difference is that cyanobacteria, unlike eukaryotes, do not produce apoptotic bodies whilst undergoing cell death. Moreover, autophagy-like parameters are shared between animal [38] and cyanobacterial cells, and cyanobacterial autophagy is similar to vacuolar cell death events in plants [39]. We propose that autophagic-like cyanobacterial cell death resembles that induced by macro-autophagosomes, which degrade the cargo of dead animal cells, and vacuolar cell death in plant cells [36]–[39]. Self-consumption, or bacterial consumption, of cyanobacterial remains may also exist in the *Azolla* symbiosis. There seem to be few differences between necrosis-like events in eukaryotes and the cyanobacterium.

The status of the peptidoglycan layer, a key component of the cell wall of all prokaryotes, may serve as a critical indicator when assessing the living/dying status of a bacterium. Our data show that retention of the peptidoglycan layer until a late stage of cell death is indicative of the apoptotic-like death mode, disappearance at an early stage is indicative of the autolytic-like mode, while maintenance at an early stage and disappearance at a late stage is indicative of the autophagic-like mode. The necrotic-like mode is characterized by an early rupture of the peptidoglycan layer. Examining 150 cyanobacterial cells showed that changes in the peptidoglycan layer positively correlated with the cell death mode in action. The peptidoglycan layer, which is composed of murein, is likely to be degraded by the murein hydrolase identified (*N*-acetylmuramyl–L-alanine–amidase) [7], which may be contained in the vesicles [17] formed at the site of the cyanobacterial peptidoglycan layer during cell death.

Cyanobacteria living within the *Azolla* plant are subject to adaptive forces manifested at various organization levels [15]–[20], [40], [41]. It is therefore likely that cell death restricts the cyanobacterial population size to maintain the symbiotic homeostasis [15], [16]. The data also suggest that PCD-like cell death events are operative during the unique vertical transfer of cyanobacteria to the next plant generation via the megasporocarp, again by trimming the population, and as earlier shown, the genome size [18]. PCD may also serve as a defense mechanism for the cyanobacterium against the host plant defense system. The cell death pathways identified here in a contemporary cyanobacterium may have existed early during the evolution of the cyanobacterial phylum. This is interesting, considering the fact that plant chloroplasts arose from a free-living cyanobacterium, and possibly from one with a complex morphology as the *Azolla* symbiont [42]; and that mitochondria, now functioning as contemporary regulators of metazoan PCD, also arose from prokaryotes [43].

Taken together, our data provide structural and biochemical evidence for the existence of four PCD or PCD-like modes in a prokaryotic cyanobacterium (plant endosymbiont) potentially genetically based in metacaspases, and show features similar to cell death pathways in eukaryotes [24] (Table 1). While the PCD-like events identified here may not represent all prokaryotes, they clearly expand the type of death modes found in non-eukaryotes. Based on our observations, we speculate that that this potentially metacaspase-based prokaryotic form of PCD-like cell death may represent an ancient form involved in the regulation of cell proliferation and cell differentiation, although the process today seems to be limited to 57% [30] of the cyanobacteria and 18% of the prokaryotes [11]. Given that chloroplasts evolved from cyanobacteria, it is also tempting to speculate that chloroplasts (together with mitochondria) may play a role as sensors and regulators in PCD processes in plant cells. Further experimental evidence is now warranted to definitively prove the operation of active cell death programs in cyanobacteria.

## Materials and Methods

### Plant Material and Growth Conditions

*Azolla microphylla* Kaulfus (IRRI No. 4018), colonized by heterocystous filamentous cyanobacteria (belonging to Section IV filamentous and heterocystous cyanobacteria; Nostocales), were grown in a greenhouse pond (Department of Ecology, Environment and Plant Sciences, Stockholm University) under a 16-h light/8-h dark cycle at 25°C with an irradiance of 550 µmol m^−2^ s^−1^. The water temperature was 20–25°C and air humidity 70–80%.

### Abiotic Stress Induction

The *Azolla* symbiosis was stressed in the following ways: *Azolla* plants were transferred to pots (15-cm diameter) containing a medium described by Watanabe et al. [44] and subjected to darkness for 5 days prior to analysis of the cyanobacteria by bright field light microscopy (LM) and transmission electron microscopy (TEM) and to nutrition deprivation by cutting off the root systems and floating the plants on distilled water for 15 days. Cyanobacteria in cavities of leaf no. 13 were then examined using LM combined with Annexin V-EGFP label, which detects cellular PS exposure (see below). Plants were also subjected to gamma (γ) radiation by placing fronds in petri dishes transparent to 40 and 80 Gy γ-radiation at a dose rate of 1.5 Gy/min generated by a ^60^Co source (Radiation Technique Center, Rice Research Institute, Fujian Academy of Agricultural Sciences, Fuzhou, China). After γ-ray exposure, plants were sampled and processed for LM and TEM (see details below).

### Light Microscopy (LM)

The cyanobacterial filaments from leaf cavities of different developmental stages were picked with a fine needle and gently spread on microscope slides coated with poly-lysine (Sigma, USA). To isolate the cyanobacterial filaments within *Azolla* sporocarps, a cover slip was placed over the sporocarp, and lightly tapped with a fine needle. The released filaments were smeared uniformly on a slide. The morphology of the cyanobacterial cells was examined using bright field and fluorescence microscopy (Olympus BX60) with filter sets for UV light and blue and green light. Exposure times were 1/15–20 s for bright field, 1/100–110 s for green light (band pass (BP) 545–580 nm), 1.2–1.8 s for blue light (BP 460–490 nm) and 1/1.2–1.8 s for UV (BP 330–385 nm).

### Transmission Electron Microscopy (TEM)

*Azolla* fronds or individual leaves and sporocarps of different developmental stages were processed for TEM according to Zheng et al. [17]. The ultrathin sections obtained were examined using transmission electron microscopy (Zeiss EM-906 or JEOL 100CX) set at an accelerating potential of 80 kV.

### Annexin V-EGFP Staining

Detection of PS was performed using the Annexin V-EGFP Apoptosis Detection Kit (BioVision, CA, USA). Based on specific features of the cyanobacterial cell types examined, the protocol recommended by the manufacturer was modified as follows: isolated cyanobacterial filaments with heterocysts and proakinetes (both with thick cell wall envelopes) were suspended in 20 µl of 1× binding buffer for 4 min with gentle mixing to prevent filament clumping. Five microliters of Annexin V-EGFP was added and the cyanobacteria were incubated in darkness in a humid chamber at room temperature for 30 min, followed by three washes in distilled water and transfer to microscopy slides, along with a drop of ProLong Antifade Reagent (Molecular Probes, Oregon, USA). Samples were examined by fluorescence microscopy (as above) using the filter set for blue light (BP 460–490 nm).

### The TUNEL Assay

To detect DNA fragmentation in cyanobacteria, the terminal deoxynucleotidyl transferase dUTP nick end labeling (TUNEL) assay was performed using the *In Situ* Cell Death Detection Kit (Roche Diangnostics, CA, USA), according to the manufacturer’s instructions, but with the following modifications: cyanobacterial cells from leaf cavities and developing sporocarps were fixed in freshly prepared 4% paraformaldehyde (PFA) in phosphate-buffered saline (PBS) for 10 min, followed by two washes in PBS. The fixed cells were made permeable by incubation in lysozyme (20 mg ml^−1^ with 100 mM EDTA in PBS) at 37°C for 60 min and were subsequently rinsed twice in PBS. Before labeling, the cells were incubated in Proteinase K (20 mg ml^−1^ in 10 mM Tris/HCl [pH 8.0], 50 mM EDTA) at 37°C for 20 min, followed by 0.1% Triton X-100 at 4°C for 4 min, to facilitate the entry of TdT into the cells. After two rinses in PBS, the samples were labeled with the TUNEL reaction mixture according to the manufacturer’s instructions. Samples were incubated in a humidified (100%) chamber in darkness. As a positive control, permeabilized cyanobacterial cells were incubated with DNase I (1 U ml^−1^) for 10 min at room temperature. A negative control was obtained by omitting TdT from the reaction mixture. DNA fragmentation was examined directly after the TUNEL reaction using fluorescence microscopy (as above) with filter sets for blue light (BP 460–490 nm).

### FITC-phalloidin Assay

To visualize the cytoskeleton of the cyanobacterium, cells were isolated from *Azolla* fronds (greenhouse grown) and placed on glass slide, fixed with 3% formaldehyde and washed with PBS. This was followed by decolorization of the cells in a series of acetone (50%–100% in PBS, each for 2 min) and washing 2 times in PBS. The cells were then incubated in 5 µl FITC-phalloidin (Alexa Fluor® 488, Invitrogen, USA) diluted 1∶10 in 40 µl buffer (0.9 M NaCl, 20 mM Tris/HCl [pH 7.5] and 0.1% (w/v) SDS), 2,5 µl 1% Triton X-100 and 2.5 µl 3% formamide, and kept at room temperature for at least 30 min. After washing with ddH_2_O two times, the cells were then stained with 50 µl DAPI (2 µg ml^−1^ DAPI in PBS) at room temperature for 2 h and then washed two times with ddH_2_O. The edges of the cover slip were sealed with nail-polish prior to fluorescence LM-examinations as above.

### DNA Extraction

*Azolla* plants were harvested, the roots removed and the plants rinsed three times with tap water. Cyanobacterial filaments were isolated as described by Ran et al. [18], with the following modifications: the cell suspension was centrifuged first in 40% Percoll (GE Healthcare, Germany) for 5 min at 360×g. The cell pellet was then re-suspended in 80% Percoll for 15 min at 1200×g, rendering a ∼95% enriched cyanobacterial cell suspension, as evidenced by light microscopy. Next, DNA was extracted from the isolated cyanobacterial cell material using a genomic DNA Extraction Kit (BioFlux, Beijing, China), with the following modifications of the manufacturer’s protocol: the sample was diluted in buffer A before all other reagents were added. Proteinase K (20 mg ml^−1^, Promega, WI, USA) was included in the solution at step 2, followed by incubation at 37°C for 1 h and at 56°C for 30 min. Extracted DNA was purified with PEG 8000 and stored at −20°C until used.

### PCD-related Genes

The p20 subunit of caspase was amplified from the isolated cyanobacteria using the primer pairs cas202, 5′-TCCCGCTTGCTGATTTTC-3′/5′-TTTGATTCACCGCTTTGG-3′ and cas203, 5′-CATTAAATATATCGGGCGAG-3′/5′-ATGAAGATGGTAAAGCAGTC-3′, and the peptideglycan hydrolase gene was amplified using the primer pair Hcw3/4 5′- GTGTTGGGCAGTTTGACG-3′/5′-ACGGCTGACACCATAACG-3′. The amplified fragments were cloned into the pGEM-T vector (Promega, WI, USA) and transformed into *Escherichia coli* DH5α using standard procedures. Gel-purified PCR products were cloned using the Cloning Kit for Sequencing (Invitrogen, USA). Nucleotide sequencing was performed with the ABI PRISM™ Dye Terminator Cycle Sequencing Ready Reaction Kit (Applied Biosystem, Beijing, China) and the reactions were analyzed on an ABI PRISM 377 DNA sequencer. The inserts were sequenced using SP6 and T7 promoter primers.

### Sequence Similarity and Alignments

To identify cyanobacterial genes and proteins that are implicated in PCD, PCR products and their translated sequences were used as queries in BLASTN and BLASTP searches, respectively, against the non-redundant (nr) database (Dec 6, 2011) with E-value cut off = 0.01. The multiple sequence alignment for ACM79336 was guided by the seed alignment of the PF00656 domain using Muscle (v3.8.31) [45], with default settings, and gaps were manually adjusted in Jalview (v. 2.7) [46].

### Accession Numbers

Sequence data from this article can be found in the GenBank/EMBL data libraries under accession numbers FJ541183 and FJ541186 and deduced amino acid accession numbers ACM79334 and ACM79336, which correspond to peptidoglycan hydrolase and the p20 subunit of caspase, respectively.

## Supporting Information

Figure S1 **Cyanobacterial colonization of developing leaves at the apex of** ***Azolla microphylla*** **.**
(TIFF)

Figure S2 **Development of the** ***Azolla*** **mega- and microsporocarps.**
(TIFF)

Figure S3 **TEM micrograph illustrating the mixture of live and dead cyanobacterial cells within a developing megasporocarp.**
(TIFF)

Figure S4 **TEM micrograph illustrating cell death modes in heterocysts and vegetative cells.**
(TIFF)

Figure S5 **Darkness enhanced cyanobacterial cell death.**
(TIFF)

Figure S6 **γ-radiation enhanced cyanobacterial cell death.**
(TIFF)

Figure S7 **TEM micrographs of cyanobacterial cells in their final death stage.**
(TIFF)

Table S1 **Results of BLASTP search against the nr database (Dec 6, 2011) using ACM79336.1 as query.**
(XLS)

Table S2 **Results of BLASTP search against the nr database (Dec 6, 2011) using ACM79334 as query.**
(XLS)
