# Supplementary figures and images for: Multiple Modes of Cell Death Discovered in a Prokaryotic (Cyanobacterial) Endosymbiont
Source: PLoS One. 2013 Jun 18;8(6):e66147. doi: 10.1371/journal.pone.0066147 (PMC3688857; doi:10.1371/journal.pone.0066147)

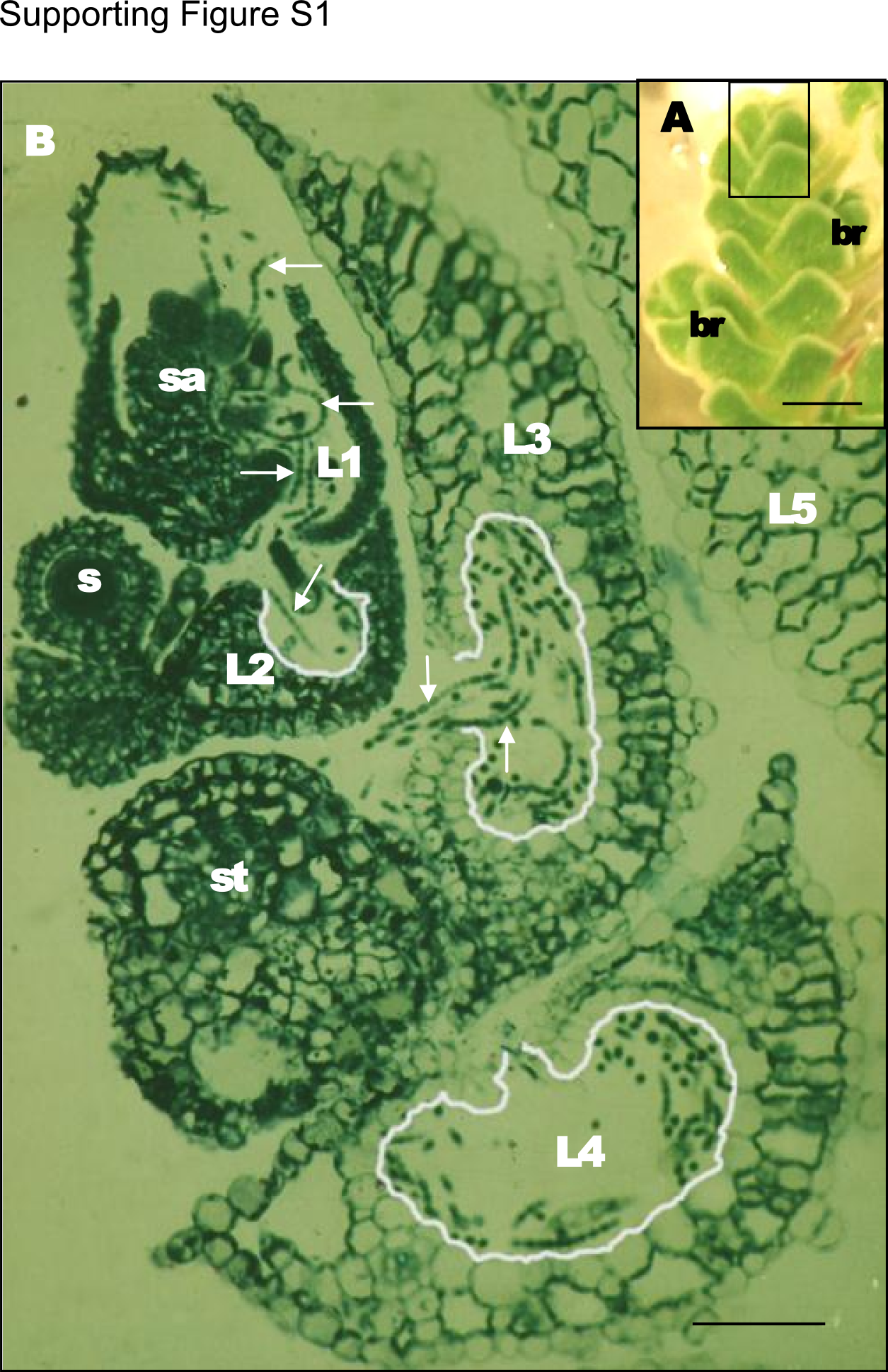

Supplement: Figure S1 — Cyanobacterial colonization of developing leaves at the apex of Azolla microphylla . (TIFF) [file pone.0066147.s001.tiff]

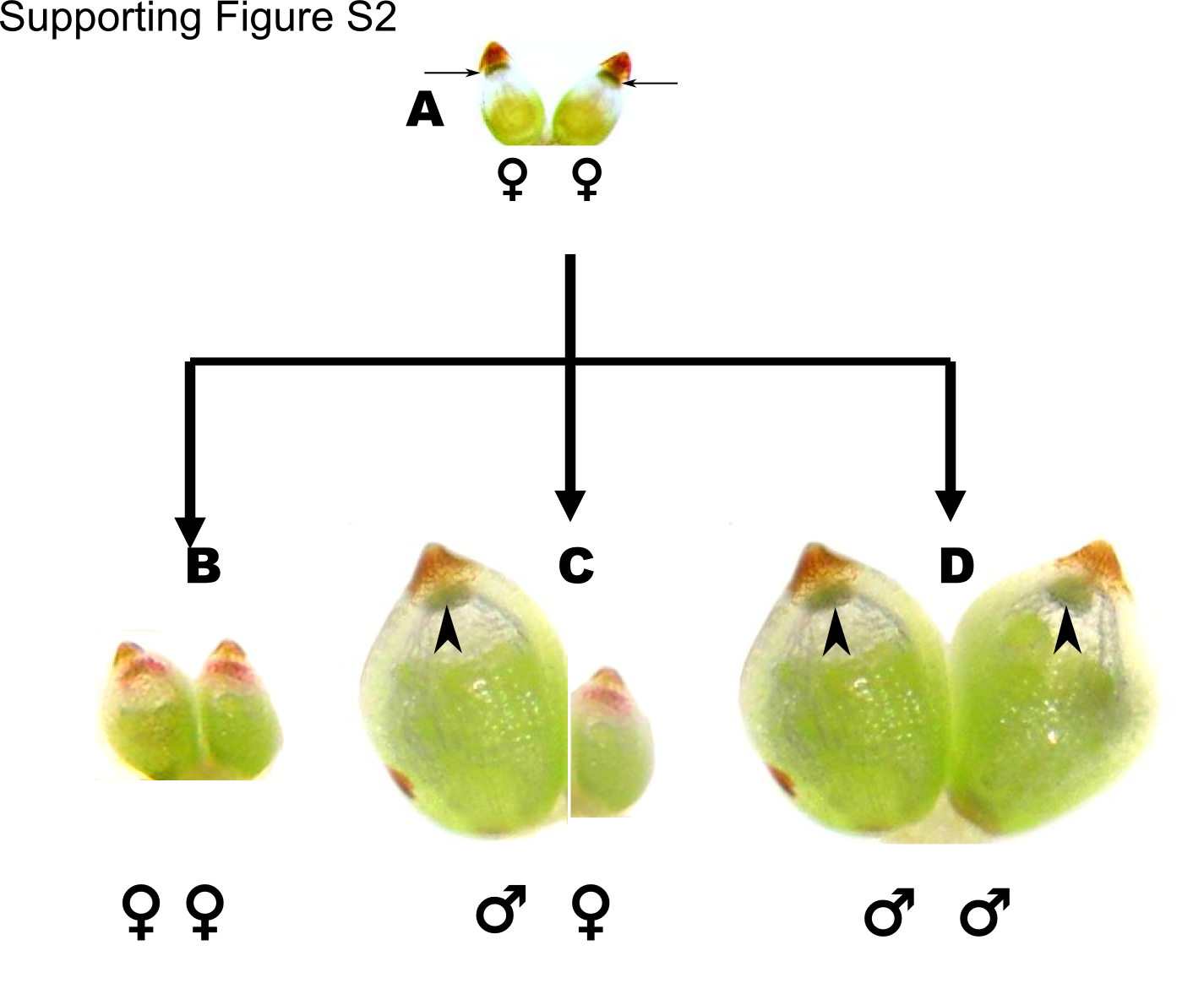

Supplement: Figure S2 — Development of the Azolla mega- and microsporocarps. (TIFF) [file pone.0066147.s002.tiff]
